# Fermented Fruits and Vegetables of Asia: A Potential Source of Probiotics

**DOI:** 10.1155/2014/250424

**Published:** 2014-05-28

**Authors:** Manas Ranjan Swain, Marimuthu Anandharaj, Ramesh Chandra Ray, Rizwana Parveen Rani

**Affiliations:** ^1^Department of Biotechnology, Indian Institute of Technology Madras, Chennai, Tamil Nadu 600036, India; ^2^Centre for Tuber Research Institute, Bhubaneshwar, Orissa 751019, India; ^3^Gandhigram Rural Institute-Deemed University, Gandhigram, Tamil Nadu 624302, India

## Abstract

As world population increases, lactic acid fermentation is expected to become an important role in preserving fresh vegetables, fruits, and other food items for feeding humanity in developing countries. However, several fermented fruits and vegetables products (Sauerkraut, Kimchi, Gundruk, Khalpi, Sinki, etc.) have a long history in human nutrition from ancient ages and are associated with the several social aspects of different communities. Among the food items, fruits and vegetables are easily perishable commodities due to their high water activity and nutritive values. These conditions are more critical in tropical and subtropical countries which favour the growth of spoilage causing microorganisms. Lactic acid fermentation increases shelf life of fruits and vegetables and also enhances several beneficial properties, including nutritive value and flavours, and reduces toxicity. Fermented fruits and vegetables can be used as a potential source of probiotics as they harbour several lactic acid bacteria such as *Lactobacillus plantarum*, *L. pentosus*, *L. brevis*, *L. acidophilus*, *L. fermentum*, *Leuconostoc fallax*, and *L. mesenteroides*. As a whole, the traditionally fermented fruits and vegetables not only serve as food supplements but also attribute towards health benefits. This review aims to describe some important Asian fermented fruits and vegetables and their significance as a potential source of probiotics.

## 1. Introduction

Fermented foods and beverages have heterogeneity of traditions and cultural preferences found in the different geographical areas, where they are produced. Fermentation has enabled our ancestors in temperate and cooler regions to survive during the winter season and those in the tropics to survive drought periods. Fermentation is a slow decomposition process of organic substances induced by microorganisms or enzymes that essentially convert carbohydrates to alcohols or organic acids [1]. In many instances, production methods of different traditional fermented foods were unknown and passed down to subsequent generations as family traditions. Drying and salting are common fermentation practices in the oldest methods of food preservation. Fermentation processes are believed to have been developed in order to preserve fruits and vegetables for times of scarcity by preserving the food by organic acid and alcohols, impart desirable flavour, texture to foods, reduce toxicity, and decrease cooking time [2].

World Health Organization (WHO) and Food and Agriculture Organization (FAO) recommended intake of a specific dose of vegetable and fruits in daily food to prevent chronic pathologies such as hypertension, coronary heart problems, and risk of strokes. The consumers tend to prefer the foods and beverages which is fresh, highly nutritional, health promoting and ready to eat or ready to drink [3]. Lactic acid (LA) fermentation of vegetables and fruits is a common practice to maintain and improve the nutritional and sensory features of food commodities [4–6]. A great number of potential lactic acid bacteria (LAB) were isolated from various traditional naturally fermented foods [7]. Asian traditional fermented foods are generally fermented by LAB such as* Lactobacillus plantarum*,* L*.* pentosus*,* L*.* brevis*,* L*.* fermentum*,* L. casei*,* Leuconostoc mesenteroides*,* L. kimchi*,* L*.* fallax*,* Weissella confusa*,* W. koreenis*,* W. cibaria,* and* Pediococcus pentosaceus*, which are considered as the probiotic source of the food practice. Availability of certain specific nutrients such as vitamins, minerals, and acidic nature of fruits and vegetables provides conducible medium for fermentation by LAB.

Probiotic is a relatively new word meaning “for life” and it is generally used to name the bacteria associated with beneficial effects for humans [8, 9]. Probiotics are defined as live microbial feed such as* Lactobacillus plantarum*,* L. casei*,* L. acidophilus*, and* Streptococcus lactis* which are supplemented by food that beneficially affect the host by improving its intestinal balance [10]. Several studies have shown that supplementation of probiotics to food provides several health benefits such as reduction of serum cholesterol, improved gastrointestinal function, enhanced immune system, and lower risk of colon cancer [11–15]. This review provides an overview on the current research prospects of LA fermentation of fruits and vegetables with regard to human nutrition and health.

## 2. Fermentation of Fruits and Vegetables by LAB

**Figure 1:**
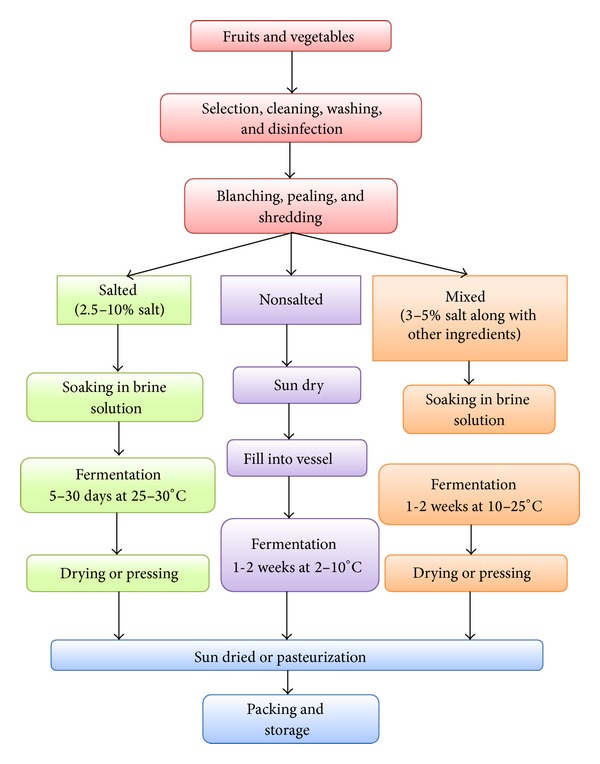
Overall fermentation process of fruits and vegetables.

Shelf life of the perishable food can be improved by fermentation which is considered as the oldest technology compared to the refrigeration. Fermentation is one of the oldest processing techniques to extend the shelf life of perishable food and was particularly important before refrigeration. LA fermentation of cabbage to produce sauerkraut has been widely studied for many years [16, 17]. Basic outline of the fruit and vegetable fermentation is given in Figure 1. With the popularity and success of sauerkraut, fermentation of many other vegetables has emerged, such as cucumbers, beets, turnips, cauliflower, celery, radishes, and carrots [18] (Table 1).

Depending on the type of raw materials in final fermented products, vegetable fermentation is characterized accordingly. Sauerkraut, fermented cucumbers, and kimchi are the most studied lactic acid fermented vegetables mainly due to their commercial importance. Canning or freezing is often too expensive method in food preservation which cannot be affordable by millions of world's economically deprived people and lactic acid fermentation [19].

Fermented fruits and vegetables (Table 2) have an important role in feeding the world's population on every continent today [20, 21]. They play an important role in preservation, production of wholesome nutritious foods in a wide variety of flavours, aromas, and textures which enrich the human diet and remove antinutritional factors to make the food safe to eat [4]. Fermentation serves many benefits, which include food security, improved nutrition, and better social well-being of the people living in marginalized and vulnerable society [22]. Fermentation-based industries are an important source of income and employment in Asia, Africa, and Latin America [23]. Fermentation of fruits and vegetables can occur “spontaneously” by the natural lactic bacterial surface microflora, such as* Lactobacillus* spp.,* Leuconostoc* spp., and* Pediococcus* spp.; however, the use of starter culture such as* L. plantarum*,* L*.* rhamnosus*,* L*.* gasseri*, and* L. acidophilus* provides consistency and reliability of performance [24].

Fruits and vegetables are exclusive sources of water-soluble vitamins C and B-complex, provitamin A, phytosterols, dietary fibres, minerals, and phytochemicals for the human diet [25]. Vegetables have low sugar content but are rich in minerals and vitamins and have neutral pH and thus provide a natural medium for LA fermentation [26]. LA fermentation enhances the organoleptic and nutritional quality of the fermented fruits and vegetables and retains the nutrients and coloured pigments [27]. LA fermentation of vegetable products applied as a preservation method for the production of finished and half-finished products is considered as an important technology and is further investigated because of the growing amount of raw materials processed in the food industry [22], and these foods are well suited to promoting the positive health image of probiotics [28]. The consumption of LA fermented fruits and vegetables helps to enhance human nutrition in several ways such as the attainment of balanced nutrition, providing vitamins, minerals, and carbohydrates, and preventing several diseases such as diarrhoea and cirrhosis of liver because of probiotic properties [29]. Some of the fermented fruits and vegetables contain coloured pigments such as flavonoids, lycopene, anthocyanin, *β*-carotene, and glucosinolates, which act as antioxidants in the body by scavenging harmful free radicals implicated in degenerative diseases like cancer, arthritis, and ageing [30]. Lactic acid fermentation of vegetables has an industrial significance only for cucumbers, cabbages, and olives [22]. In Italy, the industrial production of fermented vegetables is limited to sauerkrauts and table olives [31].

According to Kim et al. the Chinese cabbage, cabbage, tomato, carrot, and spinach provide relatively higher fermentability than other vegetables (okra and gourds) because they have more fermentable saccharides [32]. The most reported fermented fruits and vegetables are categorized as follows.

- Root vegetables: carrots, turnips, beetroot, radishes, celeriac, and sweet potato [72].
- Vegetable fruits: cucumbers, olives, tomatoes, peppers, okra, and green peas [27].
- Vegetables juices: carrot, turnips, tomato pulp, onion, sweet potato, beet, and horseradish [75].
- Fruits: apples, pears, immature mangoes, immature palms, lemons, and fruit pulps such as banana [22].

## 3. Traditional Fermented Fruits and Vegetables in India 

In eastern Himalayan regions of India a wide range of fermented vegetable products are prepared for bioprocessing the perishable vegetable for storage and further consumption [33]. Lactic acid fermentation vegetables such as gundruk, sinki, and khalpi are fermented vegetable product of Nepal, Sikkim, and Bhutan.* Lactobacillus brevis*,* L. plantarum*,* Pediococcus pentosaceus*,* P. acidilactici*, and* Leuconostoc fallax* are the predominant LAB involved in ethnic fermented vegetables. Predominant functional LAB strains associated with the ethnic fermented tender bamboo shoot products, mesu, soidon, soibum, and soijim of the Himalayas, were identified as* L. brevis*,* L. plantarum*,* L. curvatus*,* P. pentosaceus*,* L. mesenteroides *subsp.* mesenteroides*,* L. fallax*,* L. lactis*,* L. citreum*, and* Enterococcus durans* [33]. Some of the LAB strains may also possess protective and functional properties that render them as interesting candidates for use as starter culture(s) for controlled and optimized production of fermented vegetable products [34].

### 3.1. Gundruk

Gundruk is a nonsalted, fermented, and acidic vegetable product indigenous to the Himalayas. During fermentation of gundruk, fresh leaves of local vegetables known as rayosag (*Brassica rapa *subsp.* campestris *var. cuneifolia), mustard leaves (*Brassica juncea* (L.) Czern), cauliflower leaves (*Brassica oleracea* L. var. botrytis L.), and cabbages (*Brassica* sp.) are wilted for 1-2 days. Wilted leaves are crushed mildly and pressed into a container or earthen pot, made airtight and fermented naturally for about 15–22 days. After desirable fermentation, products are removed and sun-dried for 2–4 days. Gundruk is consumed as pickle or soup and has some resemblance with other fermented acidic vegetable products such as kimchi of Korea, sauerkraut of Germany, and sunki of Japan [36]. The predominant microflora of Gundruk includes various LAB such as* L. fermentum*,* L. plantarum*,* L. casei*,* L. casei* subsp.* pseudoplantarum*, and* Pediococcus pentosaceus *[33, 35].

### 3.2. Sinki

Sinki, an indigenous fermented radish tap root food, is traditionally prepared by pit fermentation, which is a unique type of biopreservation of foods by LA fermentation in the Sikkim Himalayas. For sinki production, a pit was dug with 2-3 ft diameter in a dry place. The pit is cleaned, plastered with mud, and warmed by burning. After removing the ashes, the pit is lined with bamboo sheaths and paddy straw. Radish tap roots are wilted for 2-3 days, crushed, dipped in lukewarm water, squeezed, and pressed tightly into the pit, covered with dry leaves and weighted down by heavy planks or stones. The top of the pit is plastered with mud and left to ferment for 22–30 days. After fermentation, fresh sinki is removed, cut into small pieces, sun-dried for 2-3 days, and stored at room temperature for future consumption [36]. Pit fermentation has been practiced in the South Pacific and Ethiopia for preservation of breadfruit, taro, banana, and cassava [37]. Sinki fermentation is carried out by various LAB including* L. plantarum*,* L. brevis*,* L. casei*, and* Leuconostoc fallax* [33, 38].

### 3.3. Khalpi

Khalpi or khalpi is a fermented cucumber (*Cucumis sativus* L.) product, commonly consumed by the Brahmin Nepalis in Sikkim. It is the only reported fermented cucumber product in the entire Himalayan region [36]. Ripened cucumber is cut into suitable pieces and sun-dried for 2 days, and then put into a bamboo vessel and made airtight by covering with dried leaves. It is fermented naturally at room temperature for 3–5 days. Fermentation after 5 days makes the product sour in taste. Khalpi is consumed as pickle by adding mustard oil, salt, and powdered chilies. Khalpi is prepared in the months of September and October. Microorganisms isolated from Khalpi include* L. plantarum*,* L. brevis*, and* Leuconostoc fallax* [10, 33].

### 3.4. Inziangsang

In Northeast India, especially the people of Nagaland and Manipur consume Inziangsang, traditional fermented leafy vegetable product prepared from mustard leaves and similar to gundruk [36]. Preparation process of inziangsang is like of gundruk. Mustard leaves, locally called hangam (*Brassica juncea* L. Czern), are collected, crushed, and soaked in warm water. Leaves are squeezed to remove excess water and pressed into the container and made airtight to maintain the anaerobic condition. The container is kept at ambient temperature (20°C–30°C) and allowed to ferment for 7–10 days. Like gundruk, freshly prepared inziangsang is sun-dried for 4-5 days and stored in a closed container for a year or more at room temperature for future consumption. Nagaland people consume inziangsang as a soup time with steamed rice. In resident meal, the fermented extract of* ziang dui *is used as a condiment. This fermentation is also supported by set of LAB which includes* L. plantarum*,* L. brevis*, and* Pediococcus* [10, 33].

### 3.5. Soidon

Soidonis a widespread fermented product of Manipur prepared from the tip of mature bamboo shoots. Main source of fermentation is the tips or apical meristems of mature bamboo shoots (*Bambusa tulda*,* Dendrocalamus giganteus*, and* Melocanna bambusoides*). Outer casings and lower portions of the bamboo shoots were removed and whole tips are submerged in water in an earthen pot. The sour liquid (soijim) of a previous batch is added as starter in 1 : 1 dilution, and the preparation is covered. Fermentation was carried out for 3–7 days at room temperature. Leaves of* Garcinia pedunculata* Roxb. (family: Guttiferae), locally called heibungin in Manipuri language, may be added in the fermenting vessel during fermentation to enhance the flavor of soidon. After 3–7 days, soidon is removed from the pot and stored in a closed container at room temperature for a year.* L. brevis*,* Leuconostoc fallax*, and* Lactococcus lactis* take part in fermentation [10, 39].

### 3.6. Goyang

Goyang, a prominent traditional fermented vegetable foodstuff of the Sikkim and Nepal, leafs of* magane-saag* (*Cardamine macrophylla* Willd.), belonging to the family Brassicaceae, are collected, washed, cut into pieces, and then squeezed to drain off excess water and are tightly pressed into bamboo baskets lined with two to three layers of leaves of fig plants. The tops of the baskets are then covered with fig plant leaves and fermented naturally at room temperature (15°C–25°C) for 25–30 days.* L. plantarum*,* L. brevis*,* Lactococcus lactis*,* Enterococcus faecium,* and* Pediococcus pentosaceus*, yeasts* Candida *spp., were LAB isolated from goyang [40].

## 4. Traditional Fermented Fruits and Vegetables in Other Asian Countries

### 4.1. Kimchi

Kimchi is a Korean traditional fermented vegetable made from Chinese cabbage (beachu), radish, green onion, red pepper powder, garlic, ginger, and fermented seafood (jeotgal), which is traditionally made at home and served as a side dish at meals [41]. Kimchi is a generic term indicating a group of traditional LA fermented vegetables in Korea [42]. The major raw materials (oriental cabbage or radish) are salted after prebrining, blended with various spices (red pepper, garlic, green onion, ginger, etc.) and other minor ingredients (seasonings, salted sea foods, fruits and vegetables, cereals, fish, and meats, etc.), and then fermented at low temperature (2–5°C). Kimchi fermentation is temperature-dependent process. It ripens in one week at 15°C and took three days at 25°C. But low temperature is preferred in kimchifermentation to prevent production of strong acid, overripening, and extended period of optimum taste [43]. Kimchi is characterised particularly by its sour, sweet, and carbonated taste and differs in flavour from sauerkrautand pickles that are popular fermented vegetables [44]. The classical identification of bacterial isolates from kimchi revealed that* Leuconostoc mesenteroides* and* Lactobacillus plantarum* were the predominant species [41]. Several results suggested that LAB contributing to kimchi fermentation include* L. mesenteroides*,* L. citreum*,* L. gasicomitatum*,* Lactobacillus brevis*,* L. curvatus*,* L. plantarum*,* L. sakei*,* L. lactis*,* P. pentosaceus*,* W. confusa*, and* W. koreensis* [45]. Some important species thought to be responsible for kimchi fermentation are* Leuconostoc mesenteroides*,* L. pseudomesenteroides*, and* L. lactis*, as the pH gradually falls to 4.0 [41, 42].

Kimchi contains various health-promoting components, including *β*-carotene, chlorophyll, vitamin C, and dietary fibre [43]. In addition, antimutagen [46], antioxidation, and angiotensin-converting enzyme inhibition activities of kimchi are thought to protect against disease [47]. Bacteria isolated from kimchi produce beneficial enzymes, such as dextransucrase and alcohol/acetaldehyde dehydrogenase [48]. Because of these beneficial properties, kimchi was nominated as one of the world's healthiest foods in a 2006 issue of Health Magazine [43]. Optimum taste of kimchiis attained when the pH and acidity reach approximately 4.0–4.5 and 0.5-0.6, respectively. Vitamin C content is maximal at this point.

### 4.2. Sauerkraut

Sauerkraut means sour cabbage. In sauerkraut fermentation, fresh cabbage is shredded and mixed with 2.3–3.0% salt before allowing for natural fermentation. Sauerkrautproduction typically relies on a sequential microbial process that involves heterofermentative and homofermentative LAB, generally involving* Leuconostoc* spp. in the initial phase and* Lactobacillus* spp. and* Pediococcus* spp. in the subsequent phases [42]. The pH of final product varies from 3.5 to 3.8 [49]. At this pH, the cabbage or other vegetables will be preserved for a long period of time [37]. Sauerkraut brine is an important byproduct of the cabbage fermentation industry and can be used as a substance for the production of carotenoids by* Rhodotorula rubra* or for *β*-glucosidase production by* Candida wickerhamii* for commercial applications [50].

### 4.3. Paocai

The most favored customary tableware of Chinese is Paocai, a lactic acid fermented vegetable with saltish palate. In certain places of China, the surplus vegetables such as cabbage, celery, cucumber, and radish were retained during superfluous season. Usually Paocai is served as an accompaniment with the chief meal and occasionally used as a Nipple. Paocai is a type of pickle, varies in terms of taste and method of preparation in different areas. Taiwanese paocai has crunchy texture and tangy taste, which is made with many kinds of vegetables, spices, and other ingredients by anaerobic fermentation in a special container. Paocai fermentation is initiated by various microorganisms presented in the raw materials, and LAB become the dominate bacterial finally.* Lactobacillus pentosus*,* L. plantarum*,* L. brevis*,* L. lactis*,* L. fermentum*, and* Leuconostoc mesenteroides* are the LAB isolated from paocai [36, 51].

### 4.4. Yan-Dong-Gua

In Taiwan, the extensively used customary fermented nutriment is Yan-dong-gua, prepared using wax gourd. Harvested wax gourd is washed and sliced into little pieces, dried in sunlight, combined with salt, sugar, and fermented soybeans, and layered in a bucket. Usually, minor mass of Mijiu (Taiwanese rice wine) is mixed in the earlier stage of fermentation and the bucket was sealed. The time of fermentation process is for one month, but it may be elongated even more than two months. Yan-dong-gua is usually used as a seasoning for fish, pork, meatballs, and various other foods.* Weissella cibaria* and* W. Paramesenteroides* are the bacteria responsible for fermentation [52].

### 4.5. Tempoyak

Tempoyak is a traditional Malaysian fermented condiment made from the pulp of the durian fruit (*Durio zibethinus*). Salt is sometimes added to proceed fermentation at ambient temperature. Seeded durian is mixed with small amount of salt and left to ferment at ambient temperature in a tightly closed container for 4–7 days. The acidity of tempoyak was reported as approximately 2.8 to 3.6%. The sour taste of tempoyak is attributed to the acid produced by lactic acid bacteria (LAB) during fermentation. LAB were the predominant microorganisms including* Lactobacillus brevis*,* L. mali*,* L. fermentum*,* L. durianis*,* Leuconostoc mesenteroides*, and an unidentified* Lactobacillus* sp. [53].

### 4.6. Sayur Asin

Sayur asin is a fermented mustard cabbage leaf food product of Indonesia. A similar product, hum choy, is produced in China and other South East Asian countries. Mustard cabbage leaves (*Brassica juncea* var. rugosa) are wilted, rubbed, or squeezed with 2.5%–5% salt. Liquid from boiled rice is added to provide fermentable carbohydrates to ensure that sufficient acid is produced during the fermentation. Fermentation was characterized by a sequential growth of the lactic acid bacteria,* Leuconostoc mesenteroides*,* Lactobacillus confusus*,* Lactobacillus curvatus*,* Pediococcus pentosaceus*, and* Lactobacillus plantarum*. Starch degrading species of* Bacillus*,* Staphylococcus*, and* Corynebacterium* exhibited limited growth during the first day of fermentation. The yeasts,* Candida sake* and* Candida guilliermondii*, contributed to the fermentation [54].

### 4.7. Salam Juice

Shalgam juice is prepared from the mixture of turnips, black carrot bulgur (broken wheat) flour, salt, and water by lactic acid fermentation. Shalgam is widely used in Turkey [55]. Shalgam juices were prepared by two methods for commercial production, which are the traditional and direct methods. Traditional method has two stages of fermentation that includes sour-dough fermentation (first fermentation) and carrot fermentation (second fermentation). The direct method has only second fermentation [56, 57]. The shalgam juice fermentation was mainly carried out by LAB that belong to the genera* Lactobacillus*,* Leuconostoc*, and* Pediococcus *[58, 59]. The LAB species predominantly include* Lactobacillus plantarum*,* L. brevis*,* L. paracasei*,* L. buchneri*, and* Pediococcus pentosaceus* [56, 57, 60, 61].

### 4.8. Yan-Taozih

Yan-taozih (pickled peaches) is a popular pickled fruit in China and Taiwan. Fresh peaches (*Prunus persica*) are mixed with 5%–10% salt and then shaken gently until water exudes from the peaches. The peaches are then washed and mixed with 5%–10% sugar and 1%-2% pickled plums. All of the ingredients are mixed well and then allowed to ferment at low temperature (6–10°C) for 1 day. Chen et al. isolated* Leuconostoc mesenteroides*,* L. lactis, Weissella cibaria*,* W. paramesenteroides*,* W. minor*,* Enterococcus faecalis,* and* Lactobacillus brevis* from Yan-taozih [62].

### 4.9. Pobuzihi

Pobuzihi is a widely used traditional fermented food prepared with cummingcordia in Taiwan. Two types of Pobuzihi are mainly available that can be easily differentiated from the appearance of the final products. Caked or granular pobuzihi is prepared by boiling cummingcordia (*Cordia dichotoma* Forst. f.) for several minutes and mixing it with salt. The caked pobuzihi is prepared by filling up the boiled cummingcordia into containers and after cooling removed from the containers. Chen et al. isolated novel* Lactobacillus pobuzihii*,* L. plantarum*,* Weissella cibaria*,* W. paramesenteroides*, and* Pediococcus pentosaceus* from fermented pobuzihi [63, 64].

### 4.10. Nozawana-Zuke

Nozawana-zuke is a low-salt pickle prepared by using field mustard, locally called Nozawana (*Brassica campestris* var. rapa), a leafy turnip plant. It is majorly consumed by Japanese people. The pickle is manufactured by lactic acid fermentation after adding various inorganic salts and red pepper powder containing spicy components to nozawana. The fermentation is achieved by various plant-derived genera of lactic acid bacteria (LAB), including* Lactobacillus* and* Leuconostoc. *These LAB contribute to generating the sensory properties of Nozawana zuke and preventing its contamination from disadvantageous bacteria by producing organic acids. The fermentation was carried out by* Lactobacillus curvatus* [65].

### 4.11. Yan-Jiang

Yan-jiangis a traditional fermented ginger widely used in Taiwan. It is prepared by two methods, such as with addition of plums and without addition of plums. The ginger (*Zingiber officinale* Roscoe) was washed, shredded, mixed with salt (NaCl), and layered in a bucket for 2–6 h. After the exuded water is removed, the ginger is mixed with sugar, and pickled plums are added only in method P. Salt and sugar are added to a final concentration of approximately 30–60 g kg^−1^. Fermentation usually continues for 3–5 days at low temperature (6–10°C), but some producers maintain a fermentation time of 1 week or even longer. Initial fermentation was carried out by* Lactobacillus sakei* and* Lactococcus lactis* subsp.* Lactis* and this species are replaced by* Weissella cibaria *and* L. plantarum *at the final stages of fermentation [66].

### 4.12. Yan-Tsai-Shin

Yan-tsai-shin is a fermented Broccoli (*Brassica oleracea*) stem, which is belonging to cabbage family. It is widely used in Taiwan. Harvested broccoli is washed, peeled, cut, mixed with salt (NaCl), and filled in a bucket for approximately 6 h. After the exuded water is removed, fermented broccoli is mixed with various ingredients, including sugar, soy sauce, and sesame oil. Some producers also add rice wine or sliced hot pepper to obtain a unique flavour. The ingredients were mixed well and then fermented at low temperature (6–10°C) for 1 day. The most common bacterial species include* Weissella paramesenteroides*,* W. cibaria*,* W. minor*,* Leuconostoc Mesenteroides*,* Lactobacillus Plantarum*,* and Enterococcus sulphurous *[67].

### 4.13. Jiang-Gua

Jiang-guais a popular traditional fermented cucumber in Taiwan that can be served as a side dish or a seasoning. Harvested cucumbers (*Cucumis sativus* L.) are washed, cut, mixed with salt (NaCl), layered in a bucket, and then sealed with heavy stones on the cover. This process usually continues for 4-5 h, but some producers maintain a longer processing time. After the exuded water has been drained off, the cucumbers are mixed with sugar and vinegar. In addition, soy sauce is added optionally depending on the recipe. Fermentation usually continues for at least 1 day at low temperature (6–10°C). Fermentation depends upon* Weissella cibaria*,* W. hellenica*,* L. Plantarum*,* Leuconostoc lactis*, and* Enterococcus casseliflavus* [68].

## 5. Other Fermented Vegetables and Fruits

Pickles from various vegetables and fruits such as mango (*Mangifera indica *L.) and amla (*Emblica officinalis *L.) are dietary supplements and used for culinary purposes in several parts of the world. Pickling of cucumber is made in Africa, Asia, Europe, and Latin America [69]. Khalpi is a cucumber pickle popular during summer months in Nepal [27]. Although, a variety of methods are used, placing the cucumbers in 5% salt brine is a satisfactory method. The cucumbers absorb salt until there is equilibrium between the salt in the cucumbers and the brine (about 3% salt in the brine) [70]. When the pH attains at about 4.7–5.7, the brine is inoculated with either* L. plantarum* or* Pediococcus pentosaceus* or a combination of these organisms for a total cell count of 1–4 billion cells/gallon of brined cucumbers. The final product has an acidity of 0.6–1.0% (as LA) and a pH of 3.4–3.6 in about two weeks, depending upon the temperature [71]. Similarly, sweet potato lacto-pickles may serve as an additional source of pickle with usual beneficial probiotic properties [72].

Different varieties of onions (*Allium cepa*) such as sweet, white and yellow storage were used for LA fermentation. White and yellow storage onions are typically used for processing due to their high solid content, so they were chosen for fermentation. Sweet onions are a spring/summer variety with low solids and mild flavour and are often consumed fresh.

Sweet cherry (*Prunus avium* L.) is one of the most popular of temperate fruits. Italy, together with United States, Iran, and Turkey, is one of the main world producers of sweet cherries [73].

The fermentation of beetroot and carrot juices, with addition of brewer's yeast autolysate, was also carried out by various workers like Rankin et al. A mixture of beetroot and carrot juices with brewer's yeast autolysate (fermented bio product) has optimum proportions of pigments, vitamins, and minerals. This balanced material represents a valuable product as far as nutrition and health are concerned [74]. Red beets were evaluated as a potential substrate for the production of probiotic beet juice by four species of lactic acid bacteria (*Lactobacillus acidophilus*,* L. casei*,* L. delbrueckii*, and* L. plantarum*).

Spontaneous cauliflower fermentation is commonly encountered in many countries with local variations depending mainly upon tradition and availability of raw materials.* L. plantarum* and* Leuconostoc mesenteroides* were isolated from the cauliflower fermentation [19].

The consumption of LA fermented vegetable juices (lacto-juice) has increased in many countries. Lacto-juices are produced mainly from cabbage, red beet, carrot, celery, and tomato [4]. They can be produced by either of the following procedures:

- usual way of vegetable fermentation and then processed by pressing the juice (manufacture from sauerkraut);
- fermentation of vegetable mash or juice.

There are three types of lactic fermentation of vegetable juices:

- spontaneous fermentation by natural microflora;
- fermentation by starter cultures that are added into raw materials;
- fermentation of heat-treated materials by starter cultures.

During the manufacture of lacto-juices, the pressed juice can be pasteurized at first and consecutively it is inoculated by a culture of selected LAB at a concentration varying from 2 × 10^5^ to 5 × 10^6^ CFU/mL [4, 75]. For fermentation of juices of highest quality, it is imperative to use commercially supplied starter cultures such as* L. plantarum*,* L. bavaricus*,* L. xylosus*,* L. bifidus*, and* L. brevis*. The criteria used for finding out suitability of a strain are as follows [76]:

- the rate and total production of LA, change in pH, loss of nutritionally important substances;
- decrease in nitrate concentration and production of biogenic amines (BAs);
- ability of substrate to accept a starter culture;
- type of metabolism and ability of culture to create desirable sensory properties of fermented products.

## 6. Probiotic Microorganisms

### 6.1. Lactic Acid Bacteria

The genus* Lactobacillus* is a heterogeneous group of LAB with important application in food and feed fermentation.* Lactobacilli* are used as probiotics inoculants and as starters in fermented food [77]. The genus* Lactobacillus* is Gram-positive organisms which produce lactic acid by fermentation which belongs to the large group of LAB. Other genera such as* Lactococcus*,* Enterococcus*,* Oenococcus*,* Pediococcus*,* Streptococcus*,* Leuconostoc*, and* Lactobacillus* are also considered in LAB group due to lactic acid production ability [78].

The genus* Lactobacillus* is a heterogeneous group of LAB with important implications in food and feed fermentation.* Lactobacilli* are currently used as probiotics, silage inoculants, and as starters in fermented food [77]. The genus* Lactobacillus* belongs to the large group of LAB, which are all Gram-positive organisms which produce lactic acid by fermentation. Genera of LAB include, among others,* Lactococcus*,* Enterococcus*,* Oenococcus*,* Pediococcus*,* Streptococcus*,* Leuconostoc*, and* Lactobacillus* [78].* Lactobacillus* is rod shaped, often organized in chain belonging to a large group within a family Lactobacillaceae. They grow well in anaerobic condition and strictly fermentative in nature.* Lactobacillus* is generally divided into two groups depending on the ability of the sugar fermentation: homofermentative species, converting sugars mostly into lactic acid and heterofermentative species, converting sugars into lactic acid, acetic acid and CO_2_. LAB can influence the flavour of fermented foods in a variety of ways. During fermentation, lactic acid is produced due to the metabolism of sugars. As a result, the sweetness tastes will likely decrease as sourness increases [76].

Lactobacilli prefer relatively acidic conditions ranges from pH 5.5 to 6.5 due to the main catabolite as lactic acid. It can be found in a wide ranges of ecological niches such as plant, animal, raw milks, and in insects [79]. Due to the wide verity in habitat* Lactobacillus* possess a wide range of metabolites versatility in the LAB group. It has been used for food preservation, starter for dairy products, fermented vegetables, fish, and sausages as well as silage inoculants for decades. Lactobacillus is proposed as potential probiotics due to its potential therapeutic and prophylactic attributes.* L. paracasei*,* L. rhamnosus*, and* L. casei* belong to the group of lactobacillus which are commonly found in food and feed as well as common inhabitants of the animal/human gastrointestinal tract (GIT) [80].* L. plantarum* is considered a food-grade microorganism because of its long and documented history of safe use in fermented foods [81].* L. fermentum*, one of the best-known species of this group, has been isolated from vegetable and dairy fermentation [77, 80, 82].

The* Weissella* species are Gram-positive, catalase negative, non-spore-forming, heterofermentative, nonmotile, irregular, or coccoid rod-shaped organisms [83]. Members of the genus* Weissella* have been isolated from a variety of sources, such as fresh vegetables and fermented silage [84–86]. The genus* Weissella* encompasses a phylogenetically coherent group of lactic acid bacteria and includes eight* Leuconostoc*-like species, including* Weissella confuse* (formerly* Lactobacillus confuses*),* W. minor* (formerly* Lactobacillus minor*),* W. kandleri* (formerly* Lactobacillus kandleri*),* W. halotolerans* (formerly* Lactobacillus halotolerans*),* W. viridescens* (formerly* Lactobacillus viridescens*),* W. paramesenteroides* (formerly* Leuconostoc paramesenteroides*), and* W. hellenica* [83].

### 6.2. Definition and Mechanism of Action of Probiotics

According to the Food and Agriculture Organization (FAO) Probiotics are defined as “living microorganisms which, when administrated in adequate amounts, confer health benefit on the host”. Many studies supported that maintenance of health gut microflora provides protection against gastrointestinal disorder including gastrointestinal infections and inflammatory bowel diseases. On the other hand, probiotics can be used as an alternative to the use of antibiotics in the treatment of enteric infection or to reduce the symptoms of antibiotic associated diarrhea [87]. Probiotic bacterial cultures support the growth of intestinal microbiota, by suppressing potentially harmful bacteria and reinforce the body's natural defence mechanisms. Currently, much evidence exists on the positive effects of probiotics on human health [77, 88–91].

### 6.3. Selection and Application of Probiotics

**Figure 2:**
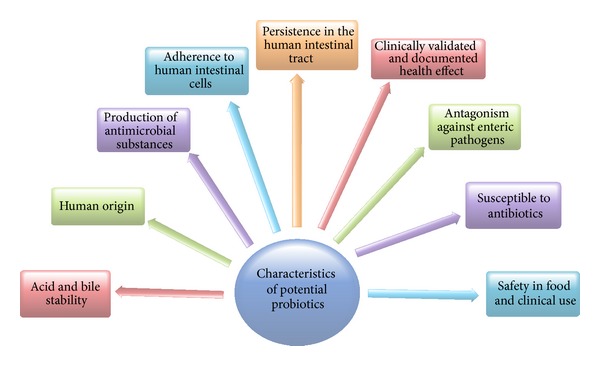
Basic characteristics of selection of a probiotic strains.

Lactobacilli are the most extensively studied and widely used probiotics within the LAB. Most* Lactobacillus* strains belong to the* L. acidophilus* group.* L. paracasei*,* L. plantarum*,* L. reuteri*, and* L. salivarius*, which represent the respective phylogenetic groups, are known to contain probiotic strains. In order for a probiotic to be of benefit to human health, it must fulfil several criteria (Figure 2). It must survive passage through the upper GIT and reach its site of action alive, and it must be able to function in the gut environment. The functional requirements of probiotics include tolerance to human gastric juice and bile, adherence to epithelial surfaces, persistence in the human GIT, immune stimulation, antagonistic activity toward intestinal pathogens (such as* Helicobacter pylori*,* Salmonella* spp.,* Listeria monocytogenes*, and* Clostridium difficile*), and the capacity to stabilize and modulate the intestinal microbiota [88–92].

## 7. Raw Materials Pretreatments

Pretreatments can promote growth of lactic flora that can be used depending on the fruit or vegetable to be fermented. Washing fruits and vegetables prior to fermentation reduces the initial microbial count, thus favouring the development of lactic flora [93]. Vegetables are also macerated with pectinolytic enzymes [75] to allow for their homogenization prior to lactic fermentation, mainly for the production of cocktails and juices [4]. Many vegetables contain glycosides that hamper efficient fermentation [94]. For LA fermentation of tomatoes, choosing very ripe fruit is recommended, since the high solanin content of unripe fruit might inhibit the growth of LAB.

## 8. Role of Ingredients Used in Fermentations of Fruits and Vegetables

### 8.1. Addition of Salt

LA fermentation of fruits and vegetables is mostly carried out in a salted medium [95]. Salting is done by adding common dry salt (NaCl) with high water content or by soaking in brine solution. The optimum salt concentration depends on the type of vegetables or fruits [96]. Substituting NaCl by KCl up to 50% in the preparation of* kimchi* from cabbage did not affect the sensory qualities (saltiness, bitterness, sourness, hotness, and texture). The main role of salt is to promote the growth of LAB over spoilage bacteria and to inhibit potential pectinolytic and proteolytic enzymes that can cause vegetable softening and further putrefaction. Salt induces plasmolysis in the plant cells and the appearance of a liquid phase, which creates anaerobic conditions around the submerged product. Anaerobic conditions are more effective in the finely cut and shredded cut material.

### 8.2. Ingredients Favouring Bacterial Growth

Some ingredients when added to LA fermented vegetables or fruits seem to enhance the development of lactic flora. They have three major roles:

- they are a source of nutrients such as sugars, vitamins, and minerals which initiate fermentation;
- they add desirable aroma, flavour, and taste to the fermented product;
- they help in combating the spoilage bacteria by lowering the pH.

For some vegetables with low nutrient contents, such as turnip and cucumber, the addition of sugar promotes bacterial growth, thereby accelerating fermentation. In Spanish-style olive fermentation, olives have undergone alkaline treatment to eliminate their bitterness, followed by repeated washings. They are then replaced with glucose on sucrose to improve LA fermentation [71]. Whey is often recommended for use in traditional LA vegetable fermentation processes as it has high lactose content, which is a potential energy substrate for LAB. It also supplies minerals salts and vitamins necessary for the lactic flora metabolism.

### 8.3. Ingredients with Antiseptic Properties

Spices or aromatic herbs are added to most of the lactic fruits and vegetable fermentation to improve the flavour of the end products [21]. Certain spices, mainly garlic, cloves, juniper berries, and red chillies help to inhibit the growth of spoilage bacteria [22]. There are many sulphur compounds with antibacterial properties in garlic which must be combined with other vegetables at ratios not higher than 150 g/kg of vegetables. Chemical preservatives such ascorbic on benzoic acid salts are sometimes used in industrial production of LA fermented* sauerkraut*, olives, or cucumbers [69]. The role of essential spice oils such as thyme, sage, lemon, and dill is to inhibit the growth during fermentations of olives [70]. Mustard seed contains allyl isothiocyanate, a volatile aromatic compound with antibacterial and antifungal properties, which inhibits the growth of yeast (*Saccharomyces cerevisiae*) and promotes growth of LAB [69, 70].

### 8.4. Ingredients Modifying the pH and Buffers Effect of Brines

To promote the growth of LAB over yeasts, moulds and other pathogenic or unwanted bacterial strains, acids, or buffer systems (acid + acid salts) are often added to the fermentation medium. During the fermentation of fruits and vegetables with high fermentable sugar contents, the fermentation medium has to be buffered to slow down acidification, thus allowing the LAB to consume all the sugars. An acetic acid + calcium acetate buffer system has been reported to improve the LA cucumber fermentation process.

## 9. Beneficial Effect of Fermented Fruits and Vegetables

### 9.1. Enhancing Food Quality and Safety

Nutritional quality of food can be enhanced by fermentation, which may improve the digestibility and beneficial components of fermented food. The raw materials have increased the level of vitamin and mineral content compared to its initial content. Several antimicrobial compounds such as organic acids, hydrogen peroxide, diacetyls, and bacteriocins are produced during the fermentation process, which impacts unrequited bacterial growth and on the other hand increases the shelf life of the food.

Lactic acid content of fermented food product may enhance the utilization of calcium, phosphorus, and iron and also increase adsorption of iron and vitamin D. Fermented foods have a variety of enzymes and each enzyme can play a different role in increasing food quality. Lactase in fermented food product degrades the lactose into galactose. Galactose is an important constituent of cerebroside that can promote brain development in infants. Similarly proteinases produced by LAB can break down the casein into small digestible molecules. Fermented foods are rich in globular fats which can be easily digested.

### 9.2. Removal of Antinutrient Compounds

Most of the fruits and vegetables contain toxins and antinutritional compounds. These can be removed or detoxified by the action of microorganisms during fermentation process. Plant foods contain a series of compounds, collectively referred to as antinutrients, which generally interfere with the assimilation of some nutrients and in some cases may even confer toxic or undesirable physiological effects. Such antinutrients include oxalate, protease, and *α*-amylase inhibitors, lectins, condensed tannins, and phytic acid. Numerous processing and cooking methods have been shown to possibly reduce the amount of these antinutrients and hence their adverse effects. It has been concluded that the way food is prepared and cooked is equally important as the identity of the food itself. Research is currently focused on identifying the antinutrient effect of several constituents rather than studying their fate during lactic acid fermentation.

### 9.3. Improving the Health Benefits of Humans

**Figure 3:**
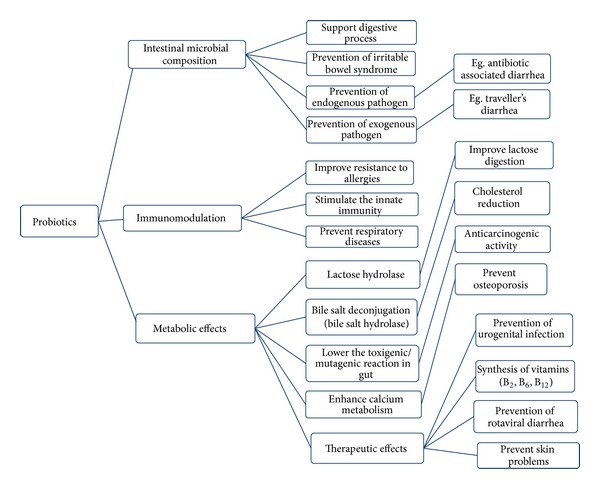
Beneficial effects of probiotics.

Several researchers have described the beneficial effects of LAB. This can modify the intestinal microbiota positively and prevent the colonization of other enteric pathogens. LAB strains also improve the digestive functions, enhance the immune system, reduce the risk of colorectal cancer, control the serum cholesterol levels, and eliminate the unrequired antinutritional compounds present in food materials. The overall health benefits of LAB are elucidated in Figure 3.

### 9.4. Biopreservation

Nowadays, consumers are particularly aware of the health concerns regarding food additives; the health benefits of “natural” and “traditional” foods, processed with no added chemical preservatives, are becoming more and more attractive. Chemical additives have generally been used to combat-specific microorganisms. In the case of fermented foods, lactic acid bacteria (LAB) have been essential for these millennia. LAB play a defining role in the preservation and microbial safety of fermented foods, thus promoting the microbial stability of the final products of fermentation. Protection of foods is due to the production of organic acids, carbon dioxide, ethanol, hydrogen peroxide, and diacetyl antifungal compounds such as fatty acids or phenyllactic acid, bacteriocins, and antibiotics such as reutericyclin [97].

The term “bacteriocin” was coined in 1953 to define colicin produced by* Escherichia coli*. Like LAB, also bacteriocins have been consumed for millennia by mankind as products of LAB and, for this reason, they may be considered as natural food ingredients. As reported by Cotter et al. “bacteriocins can be used to confer a rudimentary form of innate immunity to foodstuffs.” Bacteriocins are ribosomally synthesised, extracellularly released low molecular-mass peptides or proteins (usually 30–60 amino acids), which have a bactericidal or bacteriostatic effect on other bacteria, either in the same species (narrow spectrum) or across genera (broad spectrum) [97–99]. Bacteriocin production has been found in numerous species of bacteria, among which, due to their “generally recognized as safe” (GRAS) status, LAB have attracted great interest in terms of food safety. In fact, LAB bacteriocins enjoy a food grade and this offers food scientists the possibility of allowing the development of desirable flora in fermented foods or preventing the development of specific unwanted (spoilage and pathogenic) bacteria in both fermented and nonfermented foods by using a broad- and narrow-host-range bacteriocins, respectively.

Regarding the application of bacteriocin-producing starter strains in food fermentation, the major problem is related to the in situ antimicrobial efficacy that can be negatively influenced by various factors, such as the binding of bacteriocins to food components (fat or protein particles) and food additives (e.g., triglyceride oils), inactivation by proteases or other inhibitors, changes in solubility and charge, and changes in the cell envelope of the target bacteria [97, 100]. The most recent food application of bacteriocins encompasses their binding to polymeric packaging, a technology referred to as active packaging. Bacteriocins have generally a cationic character and easily interact with Gram-positive bacteria that have a high content of anionic lipids in the membrane determining the formation of pores [97].

## 10. Modern Techniques Used for Analyzing Microflora of Fermented Fruits and Vegetables

In addition to traditional methods (microscopy, plate count, etc.), several modern techniques like RAPD- (Random Amplified Polymorphic DNA-) PCR (Polymerase Chain Reaction), species-specific PCR, multiplex PCR, 16s rDNA sequencing, gradient gel electrophoresis, RFLPs (Restriction Fragment Length Polymorphism), and cluster analysis of TTGE (Temporal Temperature Gradient Electrophoresis) are employed to isolate and characterize different type of LAB strains of fermented fruits and vegetables [101]. RFLPs and 16s rDNA were employed to isolate and characterize lactic acid bacteria from dochi (fermented black beans) and suan-tsai (fermented mustard), a traditional fermented food in Taiwan [102]. The isolated strains are* L. plantarum*,* Salmonella enterica*,* E. coli*,* P. pentosaceus*,* Tetragenococcus halophilus*,* Bacillus licheniformis*, and so on. Tamang [10] isolated 269 strains of LAB from gundruk, sinki, inziangsang (a fermented leafy vegetable), and Khalpi samples and studied the phenotypic characteristics of these strains by genotyping using RAPD-PCR, repetitive element PCR, and species-specific PCR techniques. The major representatives of LAB involved in these fermentations were* L. plantarum*,* L. brevis*,* P. acidilactici,* and* L. fallax*. RAPD-PCR and gradient gel electrophoresis were used to isolate* L. plantarum* strains from ben saalga, a traditional fermented gruel from* Burkina Faso*. MALDI-TOF mass spectrometry and DGGE analysis were also used to analyze the fermented vegetable samples [103]. Characterization of LAB isolates by using MALDI-TOF MS fingerprinting revealed genetic variability within highly heterogeneous species. Previous research investigated the genetic diversity of LAB isolates associated with the production of fermented Almagro eggplants using a combination of randomly amplified polymorphic DNA (RAPD) and pulsed-field gel electrophoresis (PFGE) [104].

## 11. Research Prospects and Future Applications

Even though it has been broadly verified that dairy fermented products are the best matrices for delivering probiotics, there is growing evidence of the possibility of obtaining probiotic foods from nondairy matrices. Several raw materials (such as cereals, fruits, and vegetables) have recently been investigated to determine their suitability for designing new, nondairy probiotic foods [115]. Generally existing probiotics belong to the genus* Lactobacillus*. However, few strains are commercially obtainable for probiotic function (Table 1). Gene technology and relative genomics will play a role in rapid searching and developing new strains, with gene sequencing allowing for an increased thoughtful of mechanisms and the functionality of probiotics [77, 116].

## 12. Conclusion

In Asian continent, fermented fruits and vegetables are associated with several social and cultural aspects of different races. Studies showed that fruits and vegetables may serve as a suitable carrier for probiotics. Fermented fruits and vegetables contain a diverse group of prebiotic compounds which attract and stimulate the growth of probiotics. Basic understanding about the relationship between food, beneficial microorganism, and health of the human being is important to improve the quality of food and also prevention of several diseases. The amount of food ingredients and additives in fermented foods, such as sugar, salt, and monosodium glutamate, should conform to the accepted standards established by the regulations of target markets. Mixed fermentation with high variability should be replaced by pure cultivation to achieve large-scale production. Although challenges remain, it is possible that fermented foods, handed down for many generations, will play a major role in the global food industry. Detailed studies on the microbial composition and characteristics of fermented fruits and vegetables lead to the further application.

## Figures and Tables

**Table 1:** Examples of traditional fermented fruits and vegetables, which are used in various parts of Asian subcontinent.

Fermented food product	Country	Fruit and vegetables	Other ingredients	Microorganisms	References
Burong mustala	Philippines	Mustard leaf	Rock salt	*L. brevis* *Pediococcus cerevisiae *	[105]

Ca muoi	Vietnam	Eggplant		*L. fermentum* *L. pentosus* *L. brevis *	[103, 106]

Dakguadong	Thailand	Mustard leaf	Salt	*L. plantarum *	[107]

Dhamuoi	Vietnam	Cabbage, various vegetables		*Leuconostoc mesenteroides* *L. plantarum *	[37]

Dua muoi	Vietnam	Mustard or beet	Onion, sugar, and salt	*L. fermentum* *L. pentosus* *L. plantarum* *P. pentosaceus *	[103]

Gundruk	Nepal, India	Cabbage, radish, mustard, cauliflower	No	*Pediococcus and Lactobacillus spp. *	[10, 27, 37]

Inziangsang	India	Mustard leaf	No	*L. plantarum* *L. brevis,* *Pediococcus acidilactici *	[10, 36]

Jiang-gua	Taiwan	Cucumber	Salt	*Weissella cibaria* *W. hellenica* *L. Plantarum* *Leuconostoc lactis* *Enterococcus casseliflavus *	[68]

Khalpi	Nepal	Cucumber	No	*L. plantarum* *P. pentosaceus *	[10, 27]

Kimchi	Korea	Cabbage, radish, various vegetables	Garlic, red pepper, green onion, ginger, and salt	*Leuconostoc mesenteroides* *L. brevis* *L. plantarum* *L. sakei *	[42]

Nozawana-Zuke	Japan	Turnip		*L. curvatus *	[65]

Olive	Spain, Italy	Olive	Salt	*L. plantarum* *L. brevis* *L. pentosus* *P. cerevisiae* *L. mesenteroides *	[108, 109]

Pak-Gard-Dong	Thailand	Mustard leaf	Salt and sugar solution	*L. brevis* *P. cerevisiae* *L. plantarum *	[110]

Pak-sian-dong	Thailand	Leaves of Pak-sian (Gynadropsis pentaphylla)	Brine	*L. brevis* *P. cerevisiae* *L. plantarum *	[107]

Paocai	China	Cabbage, celery, cucumber, and radish	Ginger, salt, sugar, hot red pepper	*L. pentosus,* *L. plantarum* *Leuconostoc mesenteroides* *L. brevis* *L. lactis* *L. fermentum *	[36, 51]

Pobuzihi	Taiwan	Cummingcordia	Salt	*Lactobacillus pobuzihii,* *L. plantarum* *W cibaria* *W. paramesenteroides* *P. pentosaceus *	[63, 64]

Sauerkraut	International	Cabbage	Salt	*L. mesenteroides* *L. plantarum* *L. brevis* *L. rhamnosus* *L. plantarum *	[95, 111, 112]

Sayur asin	Indonesia	Mustard, cabbage	Salt, Liquid from boiled rice	*L. mesenteroides* *L. confuses* *L. plantarum* *P. pentosaceus *	[54]

Sinki	India, Nepal, and Bhutan	Radish	No	*L. plantarum* *L. brevis* *L. fermentum* *L. fallax* *P. pentosaceus *	[10]

Soidon	India	Bamboo Shoot	Water	*L. brevis* *L. fallax* *L. lactis *	[39]

Suan-tsai	Taiwan	Chinese cabbage, cabbage, Mustard leaves	Salt	*P. pentosaceus* *Tetragenococcus halophilus *	[102, 113, 114]

Sunki	Japan	Leaves of otaki-turnip	Wild apple	*L. plantarum* *L. brevis* *P. pentosaceus* *Bacillus coagulans *	[20]

Tempoyak	Malaysia	Duriyan (Durio zibethinus)	Salt	*L. brevis* *L. mesenteroides* *Lactobacillus mali* *L. fermentum *	[53]

Yan-dong-gua	Taiwan	Wax gourd	Salt, sugar, and fermented soybeans	*W. cibaria* *W. paramesenteroides *	[52]

Yan-jiang	Taiwan	Ginger	Plums, salt	*L. sakei* *Lactococcus lactis *subsp*. Lactis* *W. cibaria* *L. plantarum *	[66]

Yan-taozih	China and Taiwan	Peaches	Salt, sugar, and pickled plums	*L. mesenteroides,* *W. cibaria,* *L. lactis subsp. lactis,* *W. paramesenteroides,* *E. faecalis,* *W. minor* *L. brevis *	[62]

Yan-tsai-shin	Taiwan	Broccoli	Sugar, soy sauce, and sesame oil	*W. paramesenteroides* *W. cibaria* *W. minor* *Leuconostoc mesenteroides* *L. Plantarum* *E. sulfurous *	[67]

**Table 2:** Nutritive values and scientific names of fruits and vegetables mostly used for lactic acid fermentation.

Common name	Nutrient composition	Botanical name	Used for	Country
Leafy vegetables				
Broccoli	Carbohydrates 6.64%, Sugars 1.7% Protein 2.82% Fat 0.37% Dietary fiber 2.6%	*Brassica oleracea* L. var. italica	Yan-tsai-shin	Taiwan
Cabbage	Carbohydrates 5.8%, Sugars 3.2% Protein 1.28% Fat 0.1% Dietary fiber 2.5 g	*Brassica oleracea *	Dhamuoi Gundruk Kimchi Paocai Sauerkraut Suan-tsai	Vietnam India Korea China International Taiwan
Chinese cabbage	Carbohydrates 3.08%, Protein 0.75% Fat 0.01% Vitamin K and Molybdenum	*Brassica rapa*, subsp. *Pekinensis *	Suan-tsai	Taiwan
Mustard leaf	Carbohydrates 4% Protein 5% Total fat 1% Dietary fiber 9%	*Brassica juncea *	Burong mustala Dakguadong Dua muoi Inziangsang Pak-Gard-Dong Suan-tsai	Philippines Thailand Vietnam India Thailand Taiwan

Root and tubers				
Beet	Carbohydrates 9.96% Sugars 7.96% Protein 1.68% Fat 0.18% Dietary fiber 2.0%			
Carrots	Carbohydrates 9.6%, Sugars 4.7% Protein 0.93% Fat 0.24% Dietary fiber 2.8%	*Daucus carota *	Kanji	India
Ginger	Carbohydrates 71.62%, Sugars 3.39% Protein 8.98% Fat 4.24% Dietary fiber 14.1%	*Zingiber officinale *Roscoe	Yan-jiang	Taiwan
Radish	Carbohydrates 3.4% Sugars 1.86% Protein 0.68% Dietary fiber 1.6% Fat 0.1%	*Raphanus sativus *	Gundruk Kimchi Paocai Sinki	India Korea China India
Turnip	Carbohydrates 5% Protein 1.5% Fat 0.9%, Dietary fiber 5%	*Brassica rapa* subsp. *Rapa *	Nozuwana-Zuke Sunki	Japan Japan

Vegetables				
Bamboo Shoot	Carbohydrates 5.2% Sugars 3% Protein 2.6% Fat 0.3% Dietary fiber 2.2% Potassium 11% Zinc 12%	*Bambusa tulda *	Soidon	India
Cauliflower	Carbohydrates 5% Sugars 1.9% Protein 1.9% Fat 0.3% Dietary fiber 2%	*Brassica oleracea *	Gundruk	Nepal
Cucumber	Carbohydrate 2.7% Protein 0.67% Fat 0.13% Dietary fiber 0.8%	*Cucumis sativus *	Jiang-gua Khalpi Paocai	Taiwan Nepal, India China
Eggplant	Carbohydrate 2% Protein 2% Dietary fiber 12% Vitamin C 3%	*Solanum melongena *	Ca muoi	Vietnam
Green onion	Carbohydrates 6% Protein 3% Fat 1% Dietary fiber 7%	*Allium wakegi *	Kimchi	Korea
Wax gourd	Carbohydrates 3% Protein 2% Fat 0.5% Dietary Fiber 7%	*Benincasa hispida *Thunb.	yan-dong-gua	Taiwan

Fruits				
Cummingcordia		*Cordia dichotoma* G. Forst.	Pobuzihi	Taiwan
Durian	Carbohydrates 27.09% Protein 1.47% Fat 5.33% Dietary fiber 3.8%	*Durio zibethinus *	Tempoyak	Malaysia
Olive	Carbohydrates 3.84% Sugars 0.54% Protein 1.03% Fat 15.32% Dietary fiber 3.3%	*Olea europaea *L.	Olive	Spain, Italy
Pak-sian		*Gynandropsis pentaphylla *	Pak-sian-dong	Thailand
Peaches	Carbohydrates 9.54% Sugars 8.39% Protein 1% Fat 0.25% Dietary fiber 1.5% Vitamin C 8%	*Prunus persica *(L.) Stokes	Yan-taozih	China and Taiwan
